# Divergent mammalian body size in a stable Eocene greenhouse climate

**DOI:** 10.1038/s41598-020-60379-7

**Published:** 2020-03-04

**Authors:** Simon J. Ring, Hervé Bocherens, Oliver Wings, Márton Rabi

**Affiliations:** 1 Department of Geosciences, University of Tübingen, Hölderlinstraße 12, D-72074 Tübingen, Germany; 2 Senckenberg Research Centre for Human Evolution and Palaeoenvironment (SHEP), University of Tübingen, Hölderlinstraße 12, D-72074 Tübingen, Germany; 3 Natural Sciences Collections (ZNS), Martin-Luther University Halle-Wittenberg, Domplatz 4, D-06108 Halle (Saale), Germany

**Keywords:** Palaeoclimate, Palaeoecology, Stable isotope analysis

## Abstract

A negative correlation between body size and the latitudinal temperature gradient is well established for extant terrestrial endotherms but less so in the fossil record. Here we analyze the middle Eocene site of Geiseltal (Germany), whose record is considered to span ca. 5 Myrs of gradual global cooling, and generate one of the most extensive mammalian Paleogene body size datasets outside North America. The δ^18^O and δ^13^C isotopic analysis of bioapatite reveals signatures indicative of a humid, subtropical forest with no apparent climatic change across Geiseltal. Yet, body mass of hippomorphs and tapiromorphs diverges rapidly from a respective median body size of 39 kg and 124 kg at the base of the succession to 26 kg and 223 kg at the top. We attribute the divergent body mass evolution to a disparity in lifestyle, in which both taxa maximize their body size-related selective advantages. Our results therefore support the view that intrinsic biotic processes are an important driver of body mass outside of abrupt climate events. Moreover, the taxonomy previously used to infer the duration of the Geiseltal biota is not reproducible, which precludes chronological correlation with Eocene marine temperature curves.

## Introduction

A relationship between climate and the mean body size of endotherms was first proposed by Bergmann^1^ on the observation that mean body size seems to correspond with latitude^2^. Today, dwarfing detected in numerous modern endotherms has regularly been attributed to anthropogenic pressures, including climate change^3,4^. The fossil record allows to test the impact of climate on body size from a deep-time perspective. The Paleogene period is of particular interest because it not only witnessed the abrupt appearance, radiation and global dispersal of most modern mammal orders but it also spanned the transition from greenhouse to icehouse Earth^5^.

Several arguments based on the fossil record have been advanced in support of a negative correlation between endotherm body size and and environmental temperature. For example, ungulates during the much hotter Eocene were on average one magnitude less massive than their extant descendants^6,7^. On shorter timescales, abrupt warming events that punctuated the early Paleogene are associated with transient dwarfing of mammals^8–10^. Here we analyze the middle Eocene site of Geiseltal, whose record is considered to span ca. 5 Myrs of gradual global cooling, and generate one of the most extensive mammalian Paleogene body size datasets outside North America.

**Figure 1:**
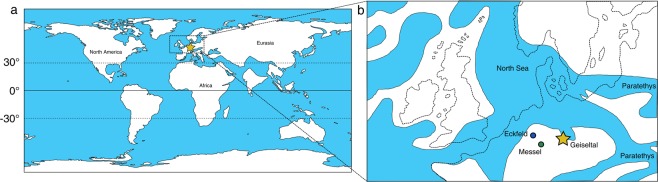
Global paleogeographic configuration (**a**) and Northern Europe (**b**) during the middle Eocene. Locations of Geiseltal, Grube Messel and Eckfeld Maar are indicated by a golden star, green circle and blue circle, respectively. The global reconstruction was created using the GPlates software (http://www.gplates.org) and the regional reconstruction was adapted from ref. ^76^.

Modern-day Germany hosts a number of internationally renowned Eocene fossil vertebrate lagerstätten, such as the Eckfelder Maar and the Messel pit (Fig. 1). However many of those sites are stratigraphically restricted and commonly span shorter time intervals of less than 1 million years^11,12^. By contrast, biostratigraphy of the coal succession at the former brown coal surface mine in Geiseltal (51°18′49″N, 11°52′08″E; Fig. 1) indicates a broadly Middle Eocene age and a temporal range of several million years of around 47.5 to 42.5 Ma for the fossiliferous sections of Geiseltal^13,14^. This makes Geiseltal a suitable target for the study of early Cenozoic environmental and ecological dynamics as this age corresponds to a period of gradual global cooling^5^. Unfortunately, despite a large vertebrate fossil volume^15^ and comparable quality of preservation^16,17^, the east German site of Geiseltal has not yet received as much scientific attention as its west German counterparts like Messel (Fig. 1).

The Geiseltal fossil lagerstätte is composed of several large coal seams that are each subdivided into between one and four fossiliferous stratigraphic horizons^18^. Coal seams are intercalated with silty and sandy grey clastic sediments in which vertebrate fossil material is largely absent. Fossil abundance is highest in the lowest section of Geiseltal and gradually declines towards the top of the profile^18^, restricting the analysis presented here to the lower coal (LC), lower middle coal (LMC), upper middle coal (UMC) and upper coal (UC). The stratigraphic interval from LC to UMC is biostratigraphically interpreted to span approximately 5 Myrs^13,14^, the ages of horizons within individual coal seams as well as the stratigraphic position of UC remain unconstrained. Fossil vertebrates in the Geiseltal strata hence provide a number of distinct geologic windows into the Eocene rather than a single continuous record.

**Figure 2:**
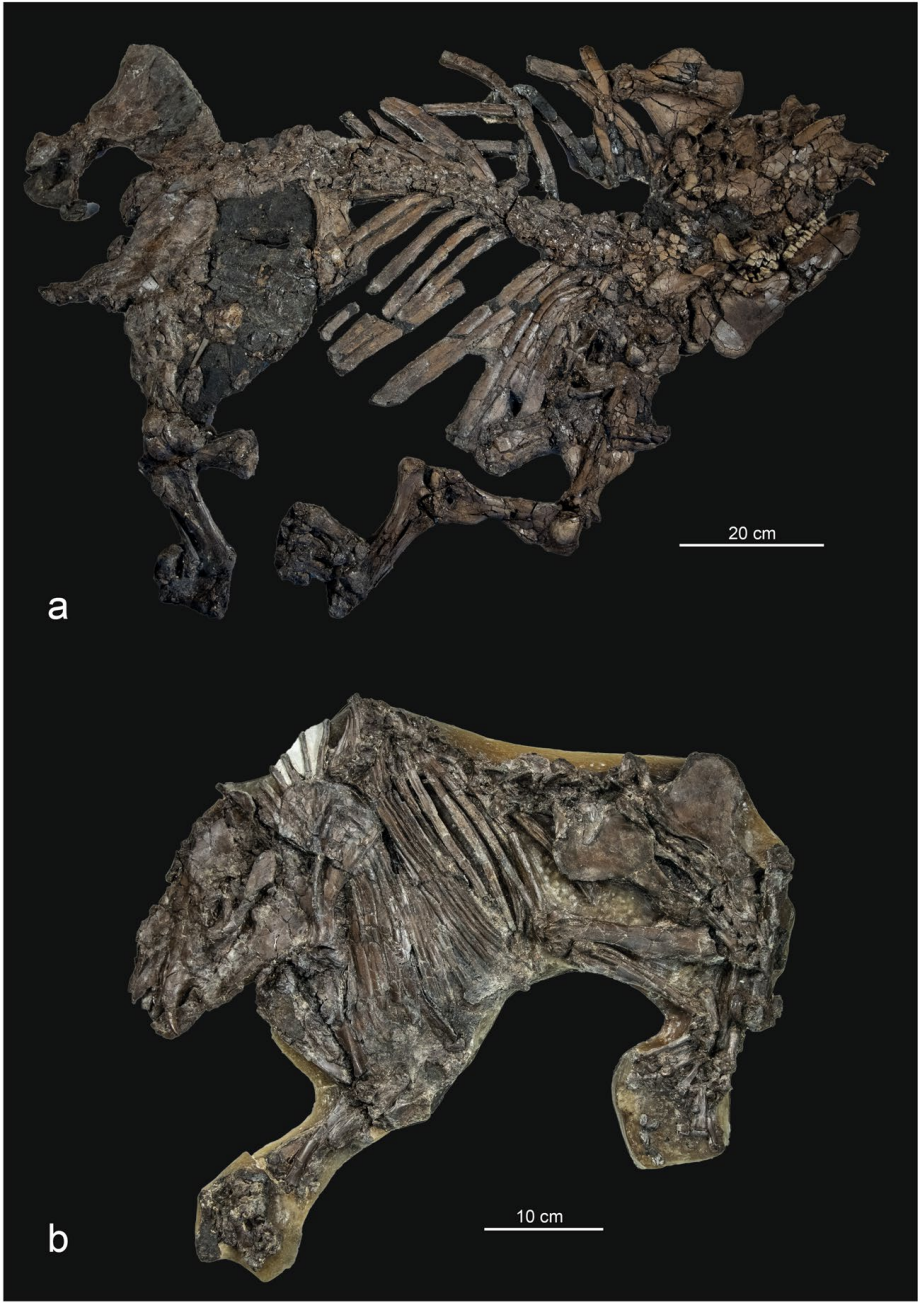
Examples of well-preserved *Lophiodon remensis* [GMH VII-1-1952] (**a**) and *Propalaeotherium isselanum* [GMH Ce IV-7011-1933] (**b**) skeletons from Geiseltal. Although fossil remains are common, complete and articulated mammal body fossils like those pictured are still rare in Geiseltal. The reconstruction of body mass therefore relies on a tooth crown proxy instead (Methods). Photographs taken by O.W.

We constructed a comprehensive analysis of changes in ungulate body mass and taxonomy across the Geiseltal profile for the tapiromorph and hippomorph taxa *Lophiodon* (Fig. 2a) and *Propalaeotherium* (Fig. 2b) - the two most abundant vertebrates at this locality. To investigate a potential coupling between dynamical change in the climate state and body size in Geiseltal, we also extracted *in-situ* oxygen and carbon isotopic measurements from the teeth of 26 individual of these two taxa.

## Results

### Taxonomic revision

Species-level distinctions in Geiseltal *Propalaeotherium* and *Lophiodon* are largely based on relative size differences in dental, cranial and autopodial characteristics^19–24^. We find that proposed diagnostic traits are non-discrete or otherwise impossible to replicate due to intraspecific variation and insufficiently well-preserved fossil material (see Supplementary Information). This indicates an artificial inflation of local mammal diversity and, as we are unable to distinguish more than a single consistent morphotype of each taxa, we here recognize only a single hippomorph and tapiromorph species at Geiseltal which we tentatively refer to *Propalaeotherium isselanum* and *Lophiodon remensis*, respectively (see Supplementary Information).

### Body mass analysis

**Figure 3:**
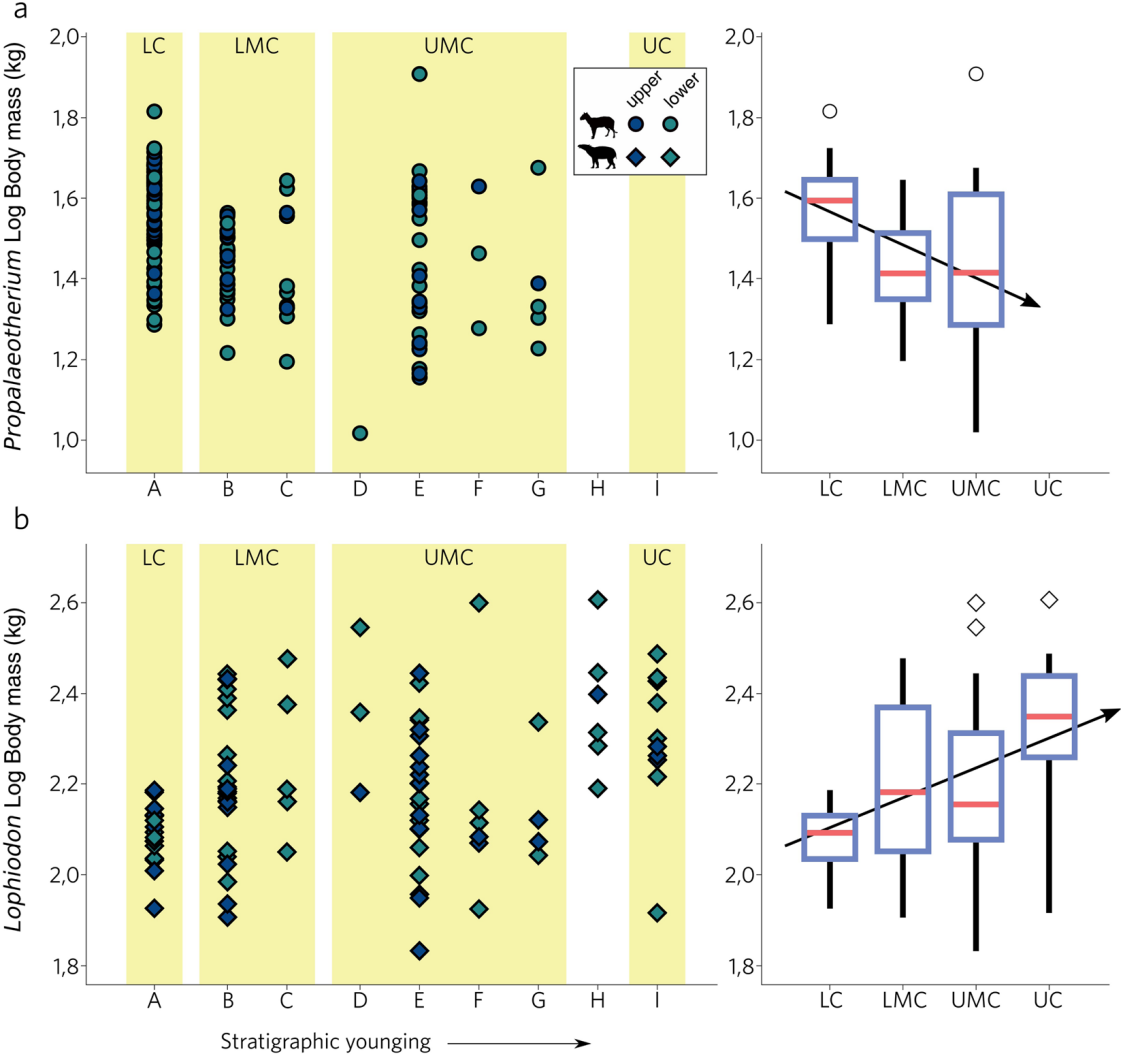
Estimated body mass of *Propalaeotherium* (**a**) and *Lophiodon* (**b**) in Geiseltal. Left column, all 257 data points, color-coded according to their position within the jaw. Right column, box-and-whiskers plot of body mass when data points are pooled together in their respective coal seams (data from Code H was included in the UC bin).

Results for *Propalaeotherium* and *Lophiodon* are given in Fig. 3 (see Supplementary Table 1). Measurements were normalized for the lower first molar (m1) because it has the closest correlation between crown area and body mass^10,25^ and results in the lowest number of total corrected data points. Estimated *Propalaeotherium* mass varies between 10.4 and 65.3 kg (Fig. 3a), excluding one outlier of >80 kg, and between 68.0 and 404.5 kg for *Lophiodon*, in broad agreement with estimates inferred from cranial dimensions^26,27^ and biomechanics^28^. The box-and-whiskers plot in Fig. 3 shows the mass distribution when our results are binned into coal seam datasets.

*Propalaeotherium* mass ranges from 19.4 to 52.8 kg (median: 39.2 kg) in LC, 15.7 to 44.0 kg (median: 25.8 kg) in the LMC and 10.4 to 47.2 kg (median: 26.0 kg) in UMC, after which the record terminates. While median body masses in the two middle coal seams are indistinguishable (*p* = 0.74 Wilcoxon-Mann-Whitney test; *p* = 0.27 two-tailed t-test), lower coal *Propalaeotherium* are significantly more massive (*p* = <0.01 Wilcoxon-Mann-Whitney test; *p* = <0.01 two-tailed t-test). Despite lower absolute abundance, the vertebrate record of *Lophiodon* is longer than that of *Propalaeotherium*, extending into UC (Fig. 3b). Interestingly, *Lophiodon* sample size in the LC (n = 16) is comparable to the relatively fossil-poor UC seam (n = 11). This is in contrast to the LC-centered general vertebrate fossil distribution in Geiseltal^18^ and may reflect a low population size of tapiromorphs in early Geiseltal. Body mass of *Lophiodon* is lowest in LC, occupying a range from 84.3 to 153.6 kg (median: 123.9 kg), compared to ranges between 80.6 and 300.0 kg (median: 152.4 kg) in the LMC, 68.0 to 278.0 kg (median: 143.0 kg) in the UMC and 82.5 to 307.2 kg (median: 223.0 kg) in the UC (including the inter-coal horizon H; Fig. 3). Statistically significant steps occur from LC to LMC (*p* = <0.01 Wilcoxon-Mann-Whitney test; *p* = <0.01 two-tailed t-test) and from UMC to UC (*p* = <0.01 Wilcoxon-Mann-Whitney test; *p* = <0.01 two-tailed t-test).

### Stable isotopic (O, C) analysis

Oxygen and carbon isotopic composition of carbonate is reported as δ^18^O_c_ and δ^13^C_c_ (where δ^18^O = ((^18^O/^16^O)_sample_/(^18^O/^16^O)_standard_ − 1) · 1.000‰ and δ^13^C = ((^13^C/^12^C)_sample_/(^13^C/^12^C)_standard_ − 1) · 1.000‰). Carbonate content in most samples falls within or slightly below the range of modern mammals^29^ and reveal no discernible correlation to the isotopic signature (Supplementary Fig. S3), indicating that exogenic incorporation of carbonate is negligible. Isotopic data are presented in Supplementary Table 2.

**Figure 4:**
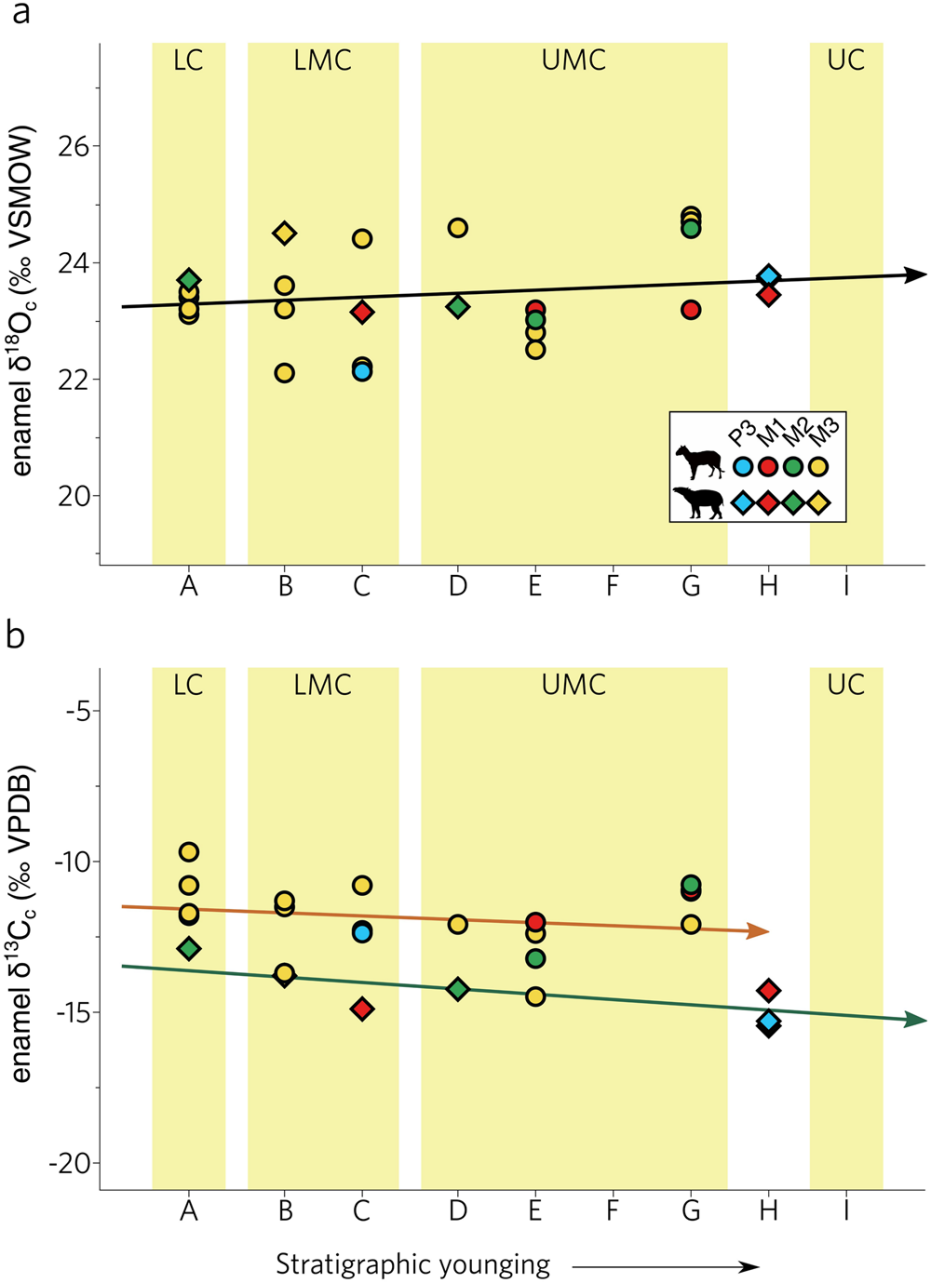
Oxygen (**a**) and carbon (**b**) isotopic results of the carbonate component in the enamel of ungulates in Geiseltal against relative stratigraphic age. Brown and green arrows represent the carbon isotopic trends for *Propalaeotherium* and *Lophiodon*, respectively.

Analysis yielded a narrow δ^18^O_c_ range from 22.1 to 24.8‰ (mean: 23.4‰; Fig. 4a). Because the third molar is last to form in the ungulate tooth eruption sequence^30^ it has been traditionally considered to be a more reliable proxy for paleoclimate reconstruction due to the reduced potential influence of weaning. However, intervals in Geiseltal where measurements from both the M3 and other tooth positions are available show very good agreement in general, supporting our contention that tooth position does not impose an important measurement bias (see Methods). Using the enamel carbonate-precipitation transfer function^31^, we compute a mean isotopic composition of precipitation (δ^18^O_p_) of between −10.8 and −7.6‰ (mean: −9.2‰). Carbon isotopic ratios (Supplementary Table 2, Fig. 4b) scatter between −13.8 and −9.7‰, with an overall mean of 12.0‰, well within the range expected for C3 plant-feeders^32^. When measurements are binned into their respective coal seams, neither isotopic system reveals a discernible change in composition with stratigraphic age (*p* = >0.05). Analytical standard deviation (1σ) is generally below 0.2‰ for the majority of samples. Within paired enamel-dentine samples (Supplementary Table 2), we find small mean offsets of only −0.4‰ and −1.5‰ for δ^18^O and δ^13^C, respectively. Considering the high susceptibility of dentine for isotopic exchange, this signals at most only very limited post-depositional diagenetic alteration^33^. Nevertheless, we find an unexpected pattern when δ^13^C paired samples are separated according to stratigraphic age. Enamel-dentine samples from the LC exhibit very minor positive offset of +1.1‰, while samples from the two middle coals have a slightly stronger negative mean offset of −2.7‰ (Supplementary Fig. S4).

## Discussion

### Middle Eocene local climate

**Figure 5:**
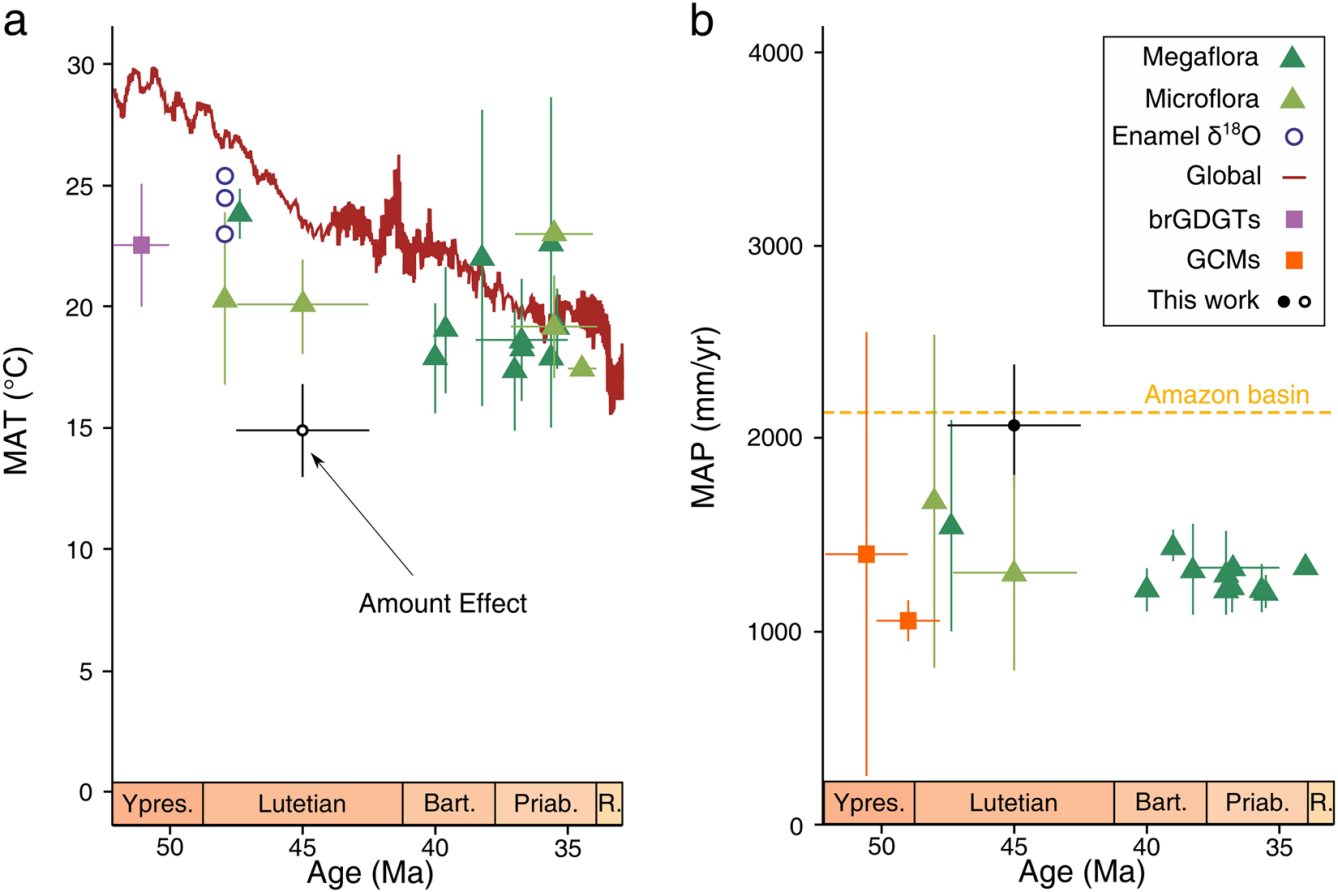
Evolution of European surface temperature and precipitation during the Eocene. When taken at face value, enamel δ^18^O-calculated surface temperature (**a**) yields anomalous values when compared to previously published proxy records (see Supplementary Information for reference list), which we interpret as an amount effect. Our δ^13^C-based MAP estimate (**b**), by contrast, is in good agreement with existing reconstructions and is comparable to modern tropical rainforests. Error bars reflect the 1σ interval.

When inferred δ^18^O_p_ values are substituted into an accepted Eocene transfer function^34^, mean annual surface temperature across all samples in Geiseltal is estimated to be 14.9 °C (±1.3 °C; 1σ). This is well below previous estimates of Central European temperature during the hot Eocene^35–39^ (Fig. 5a). Significant non-climatic overprint of tooth geochemistry is unlikely because most potential isotopic reservoirs in Geiseltal have an isotopic signature that differs markedly from our results. For example, our methodology implicitly assumes that ingested water is sourced from local precipitation. For Eocene ungulates in Geiseltal, who had a predominantly foliage-based diet and may not have been obligate drinkers, a considerable fraction of water intake might be derived from leaf water, thereby introducing a systematic bias into our data. However, because lighter oxygen isotopes are preferentially lost during evapotranspiration^40^ leaf water is characterized by a high ^18^O/^16^O ratio. Similarly, substantial post-depositional alteration is inconsistent with the close overlap between enamel and dentine and fails to account for our results because plausible alteration pathways, such as influx of dissolved carbonate ions considered to be crucial for fossil formation in Geiseltal^41^, involve δ^18^O signatures well above our results^42^. We therefore consider our results to approximate *in-vivo* conditions and attribute the anomalous oxygen isotopic composition to the ‘amount effect’ of precipitation.

The amount effect describes a decoupling between ambient temperature and isotopic rainfall chemistry due to deep moist convection, droplet re-evaporation and moisture convergence^43^ and manifests itself in an anti-correlation with precipitation intensity (i.e. δ^18^O_p_ decreases as rainfall amount increases). The amount effect is today observed at tropical latitudes in the tracks of regional summer monsoonal systems^43^. For past greenhouse intervals like the Eocene that were characterized by a expanded tropics and a shallow equator-to-pole temperature gradient^44^ it would stand to reason that this isotopic insensitivity to temperature may have extended into mid-latitudes^45^. Quantifying the relative importance of temperature and rainfall amount on isotopic chemistry during the Eocene is difficult, however, we note that our δ^18^O_p_ results are in good agreement with the composition of modelled Eocene precipitation and those inferred from organic n-alkane record of the nearby sites of Lillebaelt and Possagno^46^, indicating that the amount effect is the dominant hydrological mechanism in Geiseltal.

Carbon isotopic ratios provide a second independent way to investigate middle Eocene environment in Geiseltal. When accounted for isotopic enrichment between enamel and diet (ϵ = 14.6‰; ref. ^32^) and corrected for the different isotopic signature of middle Eocene CO_2_ (−6‰ compared to a modern value of −8‰; ref. ^47^), the isotopic composition inferred from our measurements is entirely within the range expected from C3 vegetation and signals a warm-temperate to subtropical open-canopy forest habitat^48^. For C3 plants, the fractionation between ^13^C and ^12^C is dominated by the biophysical response of plants to the availability of light and water^49^, such that δ^13^C increases with aridity. Intensity of past mean annual precipitation (MAP) can hence be constrained if the relationships between isotopic partitioning and its forcers are sufficiently well resolved. Using the quantitative relationship^50^ (assuming an absolute paleolatitude of 43° and an arbitrary low elevation of 50 m), we reconstruct Geiseltal paleo-rainfall variations of 2068 mm/yr (±319 mm/yr; 1σ) which compares favorably with Eocene general circulation model spread^51^ and proxies^35,39^ from Geiseltal coals, which indicate variability in precipitation from approx. 600 to 2500 mm/yr (Fig. 5b). Paleoclimate models agree that precipitation is generally linearly dependent on surface temperature on a global level^51^ and our isotopic measurements (both oxygen and carbon) would hence suggest no detectable long-term trend in temperature in Geiseltal, in contrast to the pronounced global cooling during the inferred time of coal deposition^5^. However, relating both variables at a single location is difficult because on a regional scale, precipitation is also sensitive to moisture convergence modulated by regional atmospheric circulation.

### Potential body size drivers

To our knowledge, the results presented here represent the most extensive investigation of Paleogene mammal physiology in Europe. Previous works that include body mass-related characteristics in Geiseltal concluded relative changes through time that are either opposite^20^ or congruent^23^ to our results - though our analysis is based on sample sizes around ten times larger.

The invariant isotope geochemistry and opposite sign of body mass change through the LC-LMC transition in both genera precludes a straightforward climatic effect analogous to early Paleogene hyperthermals, as a direct climate forcing would elicit a physiological response in the same direction. A broad similarity in body mass behavior (i.e. marked change from LC to LMC and identical mean body masses between LMC and UMC) may nevertheless indicate size-specific responses to a common driver.

Additionally, ecology places as an equally important constraint on mammal body size that is independent of climate. Because body size directly scales with many life history traits (such as reproductive rate or home range size)^52^ that determine the efficiency of resource acquisition, different dietary niches are likely associated with distinct optimal body size ranges^53^. Interaction with other organisms can additionally influence the resource use and therefore the available niche space that is open for a given taxon. For example, Smith and Lyons^54^ note that the body size of African hyraxes dropped by several orders of magnitude following the immigration of competitive herbivores during the Oligocene. Under this interpretation, the substantial increase in mammalian body mass during the Cenozoic reflects an ecological release and progressive filling of empty niche space that favors large-bodied organisms rather than the effect of an abiotic forcing like temperature^55^.

Sample sizes of *Lophiodon* are lowest when *Propalaeotherium* abundance is highest and vice versa, consistent with a changing competitive balance^56^ and conventional theory predicts that increasing competitive pressure will result in character displacement (CD) in which both competitors will tend towards minimizing their trait similarities^57^. Nevertheless, unambiguous examples of CD are rare^58^ and trade-offs in population sizes may also arise from independent dynamics of species ranges instead of CD. It is further unclear to which extent large tapiromorphs and small horses were truly subject to resource competition as we observe an offset in enamel δ^13^C values, suggestive of a body-size related difference in resource utilization, similar to other extant and fossil mammal communities^48,52^. Furthermore, a strong competitive pressure would be expected to lead a decrease in trait variance. We, by contrast, document a sharp increase in body size variance for *Lophiodon* and no pervasive variance change in *Propalaeotherium* (Fig. 3).

Alternatively, different feeding strategies and resource partitioning between larger, less forage-selective mammals and smaller, more specialized mammals could lead to divergent responses under selective pressures. According to the fast-slow life history concept^59^, small animals have a higher mortality but more rapid reproduction compared to larger animals. Therefore, a divergent body size evolution could be the result of a maximization of selective advantages as both taxa evolve in opposite directions along the fast-slow life history continuum. For example, a larger body mass could confer a larger migratory range, reduced predation pressure and more effective metabolization on *Lophiodon*, the largest herbivores of the European Eocene. On the other hand, the dwarfing of *Propalaeotherium* would result in a faster transfer of resources to reproduction per unit of body mass^60^. A progressive differentiation into their respective fast and slow lifestyles explains, in contrast to CD, why the tapiromorph body size increase persists after *Propalaeotherium* becomes rare in Geiseltal.

### Implications for the formation and placement of Geiseltal

Krutzsch *et al*.^39^ proposed that the Geiseltal coal succession resulted from a cyclical transition between episodes of persistently elevated humidity that promoted deposition of organic matter and a semi-arid climate with diminished vegetation and reduced coal formation. Though vertebrate fossils are overwhelmingly concentrated in coal-bearing sequences, few remains are preserved in the grey, unconsolidated sands between (Code H in Fig. 4), allowing for an independent evaluation. We find that the isotopic composition of enamel preserved in coal-poor strata are not substantially different from samples of other stratigraphic horizons, refuting a strong climatic control on local stratigraphy. Furthermore, because coal-forming peats are normally unconducive towards the preservation of biogenic hard tissues due to the build-up of humic acids, buffering by dissolved bicarbonate from the Muschelkalk limestone group is considered as a necessary condition for the deposition of vertebrate fossils in Geiseltal^41^. Enamel in the LC is on average depleted in ^13^C relative to dentine (above the 1:1 line where enamel δ^13^C is equal to dentine δ^13^C) while enamel from the LMC and UMC are relatively enriched in δ^13^C (below the 1:1 line in Supplementary Fig. S4). One possible, but non-restrictive, explanation is that this represents the receding influence of percolating dissolved bicarbonate which has a δ^13^C signature well above Geiseltal mammals^42^, and the increasing role of compositionally low-δ^13^C lignites^61^, potentially explaining why fossil abundance is highly concentrated at the base of Geiseltal.

Due to an absence of radiometrically datable igneous rocks, the principal means by which the age of Geiseltal is constrained is biostratigraphy. For individual coal-bearing sequences, age is inferred by comparison of its mammal composition with more well dated European localities. A similarity in reptilian^62,63^, avian^64^ and mammal faunal assemblages^18,20,65,66^ has lead to the conclusion that the Geiseltal LC is contemporaneous to the early Lutetian site of Messel, while LMC, UMC and UC have been correlated with the sites of Issel (France), Bouxwiller (France) and Egerkingen (Switzerland), respectively^14,18,20^. However, numerous traits considered to be diagnostic of Geiseltal *Propalaeotherium* species that are predominantly used in Eocene mammal biochronology are poorly defined, not reproducible or potentially within intra-specific variability (see Supplementary Information). This indicates that multiple Geiseltal palaeotheriids are subject to taxonomic over-splitting which would strongly distort biostratigraphic analyses. The deposition of lignite beds with the same minimal vertical thickness as in Geiseltal requires just over 1 Myrs, assuming the maximum present-day coal accumulation rate^67^.

In our view, this highlights the need for a reevaluation of regional biostratigraphic mammal zonation (i.e., the age of Geiseltal relative to European Eocene vertebrate localities) and indicates a temporally more restricted Geiseltal record although still likely at the Myr scale.

## Conclusions

Middle Eocene lignites in Geiseltal document a discontinuous succession of floral and faunal assemblages. Oxygen and carbon compositional *in-situ* measurement of molars support near-*in vivo* preservation and suggest that both form complementary records of intensified palaeo-precipitation, consistent with an expansion of the (sub)tropics during the Paleogene greenhouse interval. We further find little evidence of long-term change in climate, despite supposedly coeval progressive global-scale cooling. Extensive measurement of m1 crown area of the two most abundant vertebrates (*Propalaeotherium* and *Lophiodon*) indicates that both varied significantly in body size through the Geiseltal profile and exhibit an opposite directionality. We explore multiple interpretations and conclude that instead of climatic adaptation, the exploitation of relative selective advantages resulting from different life histories (‘slow’ vs. ‘fast’ living) of both species is the most plausible explanation. As large herbivorous mammals appear in Europe relatively late in the Paleogene, a promising direction of further research is to better constrain when and how these body size-related life histories first evolved compared to larger continents. We were unable to replicate most of the diagnostic traits outlined for Geiseltal hippomorphs and tapiromorphs that otherwise largely underpin the regional biochronology, which suggests that previous alignment of Geiseltal with other localities is subject to arbitrary inflation of species diversity. This highlights the need for a reexamination of current biostratigraphic correlations of both Geiseltal and the Geiseltalian European Land Mammal mega-zone with the European terrestrial mammal chronology.

## Methods

### Taxonomic revision

Mammal taxonomy is a crucial tool for determining the biostratigraphic provenance of Geiseltal and has importance for our body mass analysis. We performed an assessment of the most recent published systematic diagnoses presented in refs.^19^,^20^,^23^ and tested for re-producibility by examining holotypes and referred specimens of the species *Propalaeotherium hassiacum*, *P. isselanum*, *P. voigti*, *Lophiodon cuvieri*, *L. remensis*, *L. tapirotherium*, *Rhinocerolophiodon buchsovillanum* and *Eurohippus parvulus*, kept at the Geiseltal Collection in Halle an der Saale, Germany (see Supplementary Information).

### Body mass analysis

Complete fossil skeletons are exceedingly rare compared to the number of incomplete vertebrate remains from Geiseltal. Therefore, to largely overcome this taphonomic effect, rather than measuring anatomy directly, a variable that approximates body size is used instead. The proxy analyzed in this study exploits an ungulate-specific observational relationship between the crown area of the first molar (M1; defined as the buccal-lingual length multiplied by distal-mesial length; see Supplementary Fig. S1) and mean body mass (Eq. (1))^25^:

1 $$\mathrm{ln}({\rm{a}})=1.564\cdot \,\mathrm{ln}({\rm{b}})+3.267$$

where a is the mean body mass (in g) and b is the tooth crown area (in mm^2^). This approach has proven to be a reliable tool for body size reconstruction^25^ and has recently been used to successfully demonstrate climate-driven mammalian dwarfing during the early Eocene^10^. All measurements were taken with a digital caliper in the Geiseltal Collection in Halle an der Saale (Germany), which hosts the largest collection of Geiseltal fossil material. Because body mass can differ markedly from one species to another^20^ it is necessary to restrict analysis to fewer taxa that have a large stratigraphic range and are found in sufficient number to produce meaningful results. Therefore our analysis was restricted to 157 specimens of *Propalaeotherium*, and 100 specimens of *Lophiodon* where the m1 could be firmly identified. To reduce measurement inaccuracy, every tooth dimension was measured three times (which translates to more than 1500 individual measurements) and the respective arithmetic means were used for calculation. Furthermore, owing to differing tooth morphology between upper and lower first molar, the crown area proxy can produce discrepant results for the same individual. To avoid bias by inhomogeneous sampling of upper and lower first molars, we constructed an intrageneric correction factor for *Propalaeotherium* and *Lophiodon* using a least-squares tooth regression of fossil specimen from individuals that preserve both their maxilla and mandible. We find that tooth dimensions between upper and lower M1 are broadly correlated (see Supplementary Fig. S2) for *Propalaeotherium* (n = 9; r^2^ = 0.69) and *Lophiodon* (n = 9; r^2^ = 0.66) through the following equations:

2 $$\mathrm{ln}({{\rm{l}}}_{{\rm{P}}})=0.9144\cdot \,\mathrm{ln}({{\rm{u}}}_{{\rm{P}}})-0.0327$$

3 $$\mathrm{ln}({{\rm{l}}}_{{\rm{L}}})=0.7689\cdot {\mathrm{ln}({\rm{u}}}_{{\rm{L}}})+0.8549$$

where u and l are the respective crown areas (in mm^2^) of upper and lower M1. Subscripts P and L denote the genera *Propalaeotherium* and *Lophiodon*, respectively.

### Stable isotopic (O, C) analysis

Vertebrate skeletal hard tissues such as enamel and dentine are principally composed of a calcium-phosphate lattice (Ca_5_(PO_4_)_3_) that can contain significant substitution of carbonate (CO_3_^2−^). The isotopic composition of these fossilized biominerals is commonly considered to be a reliable proxy for paleoenvironmental reconstruction because of their refractory nature and ability for primordial geochemical preservation at geologic timescales^68,69^. While carbon isotopic values can be directly interpreted in terms of dietary and habitat preferences^58^, oxygen stable isotope values are tied to a step-wise reasoning process: because homeothermic animals have a constant body temperature, oxygen isotopic composition in bioapatite and carbonate reflect the composition of ingested water^70,71^, of which meteoric surface water is the dominant reservoir for obligate drinkers. Fractionation of meteoric water molecules moving through the hydrological cycle, in turn, is climate-dependent^72^, and can therefore be used for paleoclimate reconstructions. We sampled the isotopic composition of the carbonate component in enamel and dentine from 26 isolated *Propalaeotherium* and *Lophiodon* molars from Geiseltal. Bulk powder-samples were extracted using a micromotor drill and subsequently subjected to a pre-treatment process to remove the potentially polluting effect of secondary carbonate (see Supplementary Information). Measurements were made with an isotope ratio mass spectrometer (IRMS) at the University of Tübingen.

We do not anticipate significant intra-jaw variability of δ^18^O because of the likely accelerated enamel biomineralization of smaller, brachydont equids^73^. To obtain the isotopic composition of local paleo-surface water, we invoke the calibration between ingested water and the CO_3_^2–^component of bioapatite presented by Zanazzi *et al*.^31^:

4 $${\delta }^{18}{{\rm{O}}}_{{\rm{p}}}=1.16\cdot {\delta }^{18}{{\rm{O}}}_{{\rm{c}}}-36.4\textperthousand $$

We prefer this approach because it reduces the propagated error compared to first relating carbonate to phosphate isotopic composition before constraining paleo-precipitation chemistry. We are unaware of a carbonate-meteoric calibration for tapirs and therefore also apply Eq. (4) to our measurements of molars from *Lophiodon*, implicitly assuming that basal sections of both branches of perissodactyls (tapiromorphs and hippomorphs) had similar isotopic fractionation.

Though there is evidence for limited ice volume in the middle Eocene^74,75^, we nevertheless apply the −1‰ correction for the ice-free ocean on δ^18^O_p_ to provide consistency with previous studies.

## Supplementary information

Supplementary Information.

Supplementary Table 2

Supplementary Table 1
