# Supplementary material for: Divergent mammalian body size in a stable Eocene greenhouse climate
Source: Sci Rep. 2020 Mar 4;10:3987. doi: 10.1038/s41598-020-60379-7 (PMC7055232; doi:10.1038/s41598-020-60379-7)
Supplement: Supplementary file 1 — Supplementary Information. [file 41598_2020_60379_MOESM1_ESM.pdf]

# Supplementary to: Divergent mammalian body size in a stable Eocene greenhouse climate

Simon J. Ring[1], Hervé Bocherens[1,2], Oliver Wings[3] & Márton Rabi[1,3]

[1]Institut für Geowissenschaften, University of Tübingen, Hölderlinstraße 12, D-72074 Tübingen, Germany
[2]Senckenberg Research Centre for Human Evolution and Palaeoenvironment, University Tübingen, Hölderlinstraße 12, D-72074 Tübingen, Germany
[3]Zentralmagazin Naturwissenschaftlicher Sammlungen (ZNS), Martin-Luther University Halle-Wittenberg, Domplatz 4, D-06108 Halle an der Saale, Germany

The SI contains information on (i) pretreatment protocol applied to enamel samples during isotopic analysis, (ii) a detailed summary of Geiseltal mammal taxonomy relevant for this paper, (iii) referenced proxy estimates used to construct Figure 5, and (iv) Supplementary Figures S1–S4

**(i) Isotopic measurements**
The pre-treatment consisted of soaking each sample in 2 ml of 2.5% chlorinated sodium hypochloride (NaClO) at room temperature. After 24 hours, skeletal tissue and solution were separated by 2 minute centrifugation, after which the supernatant was manually extracted and discarded with a pipette. Samples were then subjected to three rounds of washing in distilled water and immersed in a buffer solution consisting of acetic acid ($CH_3COOH$) and sodium hydroxide (NaOH) for another 24 hours. Following another three rounds of distilled water washing, the pre-treated samples were evaporated until dryness under 37°C in an incubator. In addition to enamel and dentine, bone standards of known isotopic composition were also analyzed as a quality control. All samples were separately dissolved in phosphoric acid ($H_3PO_4$). As the analyte gas, developing $CO_2$ was subsequently measured for isotopic species $^{12}C^{16}O$, $^{13}C^{16}O$ and $^{12}C^{18}O$ in the Isotope-ratio mass spectrometer at the University of Tübingen, from which oxygen and carbon isotopic ratios were calculated.

**(ii) Systematic Paleontology**
***(ii.1) Hippomorpha***
***Valid Taxa***

PERISSODACTYLA Owen, 1848
EQUOIDEA Hay, 1902
PALAEOTHERIIDAE Bonaparte, 1850
*Propalaeotherium isselanum* Cuvier, 1824

This species was initially described from the Lutetian of France (the type is from Issel) and has been reported from Geiseltal and Eckfeld in Germany[1,2,3,4,5]. Our taxonomic revision of material from Geiseltal (see Supplementary Table 1) reveals that there is no evidence for more than a single species of palaeotheriids at this locality because the characters previously considered to diagnose more than a single species[6] are impossible to reproduce. We refer all Geiseltal specimens previously identified as *Propalaeotherium voigti*, *P. hassiacum*, *P. argentonicum* and *Eurohippus* (=*Propalaeotherium*) *parvulus* to a single species, *P. isselanum*. We consider *P. isselanum* as the proper name for the only valid species in Geiseltal because it has nomenclatural priority. However, we note that this referral is tentative as we did not study the type material of *P. isselanum*. Moreover, the holotype of *P. isselanum* is fragmentary and the type locality material of the species

requires redescription. Remy[2] proposed that the *P. isselanum* of Franzen and Haubold[6] (from Geiseltal) may not be identical to *P. isselanum* from France. We furthermore note that some of the palaeotheriid taxa reported from Geiseltal may be diagnosed at other localities. Previously, the most recent revision of these taxa was in Franzen and Haubold[6] , predominantly based on the Geiseltal material. A single lower m3 has been referred to *P. argentonicum* from the lower coal (GMH-XIV-203) by Franzen and Haubold[6] . The Geiseltal specimen is referred to *P. argentonicum* on the basis of its large size (this is the largest species of *Propalaeotherium* according to Franzen and Haubold[6]) and the more hypsodont molars. Given that (i) these differences were not quantified, (ii) the height and size of the crown falls within variation seen in Geiseltal *P. isselanum*, and (iii) the species is thought to be represented by a single specimen only, we also refer this specimen to *P. Isselanum*.

***Dubious Taxa***

*Propalaeotherium hassiacum* Haupt 1925 nomen dubium

This species was described from Messel[7] and the most recent revision of its diagnosis is Franzen and Haubold[6] based on Geiseltal material. As our reassessment reveals, none of the diagnostic characters of *P. hassiacum* proposed by Franzen and Haubold[6] are discrete or allow recognizing a distinct morphotype and we therefore refer all Geiseltal specimens of *P. hassiacum* to the species with priority, which is *P. isselanum*. Franzen and Haubold[6] considered *P. isselanum* the phyletic descendant of the stratigraphically older *P. hassiacum*, an assertion we principally agree with but, at least in Geiseltal, these taxa cannot be separated into more than a single morphospecies. Until the holotype of *P. hassiacum* (from Messel) is redescribed in greater detail, we find this taxon to be dubious, as we are unable to find characters that allow us to rigorously diagnose a valid taxon on the basis of the Geiseltal collection (which in turn formed the basis of the most recent taxonomic revision of this species[6]).

The following characters were used to distinguish *P. hassiacum* from *P. isselanum* in ref. 6 :

(a) Molars are more brachybunodont with weaker outer wall relief and weaker mesostyle than in *P. isselanum*.
*Comment*: In the absence of quantitative data these differences are impossible to reproduce and are otherwise not apparent as discrete morphotypes in the Geiseltal sample. Besides, both *P. isselanum* and *P. hassiacum* have apparent mesostyls that are practically indistinguishable in size in the two taxa.

(b) Posterolingual corner of the upper premolars (particularly P4) is noticeably retracted and the horizontal outline of the crown is hence sub-triangular.
*Comment*: The P4 is subtriangular in both taxa and some referred specimens of both *P. isselanum* and *P. hassiacum* are slightly more quadrangular/u-shaped in their horizontal outline.

(c) Postproto- and posthypocristid are more developed than in *P. isselanum*.
*Comment*: These are equally developed in both taxa and if any difference is present, it is likely due to tooth wear.

(d) The entoconid of p4 is either weaker in *P. isselanum* or completely absent.
*Comment*: The entoconid in *P. hassiacum* is often as developed as in *P. isselanum*, forming a single distinct cusp.

(e) The p1 always has two roots in *P. hassiacum*, unlike *P. isselanum* which often has only one.
*Comment*: We found no evidence for single-root p1 in the lower jaws of *P. isselanum*.

(f) The jaw is more robust than that of *P. isselanum*.
*Comment*: In fact, some specimens reffered to *P. isselanum* are more robust than *P. isselanum*. Furthermore, this character is not quantified and variability has not been assessed.

(g) Extremities are broader and shorter than that of *P. parvulum* and *P. voigti*, especially the autopods. The autopods are longer than in *P. isselanum*.
*Comment*: The Geiseltal collection of *P. hassiacum* consists of disarticulated skeletal material with unknown association and the relative body proportions of these taxa are therefore impossible to compare. For instance, no autopod of a single individual is known for *P. hassiacum* which precludes comparing its proportions to that of *P. isselanum*. Instead, using circular reasoning, larger sized postcranial elements from the lower coal has been consistently referred to *P. hassiacum* with no diagnostic features present and of unknown ontogenetic status. Moreover, potential intraspecific variation of body proportions has not been addressed by Franzen and Haubold[6].

*Propalaeotherium parvulum* Laurillard 1849 = *Eurohippus parvulus* sensu Franzen 2006

*Propalaeotherium* parvulum was recombined into *Eurohippus parvulus* by Franzen[8] in order to express his view that this species is not closely related to other species of *Propalaeotherium*. A separate species is recognized from Messel (*E. messelensis* Haupt, 1925; Franzen, 2007) whereas the few specimens from Geiseltal are being referred to *E. parvulus* (e.g., ref. 9). In the Geiseltal sample, the characters of Franzen[8] are insufficient to distinguish *E. parvulus* from *P. isselanum*. As there is no reproducible morphotype from Geiseltal that would correspond to *E. parvulus*, we assign all specimens previously identified as *E. parvulus* to *P. isselanum*. Our study also casts doubt on the validity of *E. parvulus* and *E. messelensis* (from Messel) as intraspecific variation was apparently not taken into account while diagnosing these species.

According to Franzen[8], *Eurohippus* differs from *Propalaeotherium* Gervais 1849 by
(a) smaller size
*Comment*: In the lack of studies on intraspecific differences in size (i.e. ontogeny, sex) in *P. isselanum*, size cannot be used for separating species. Moreover, the material referred to *E. parvulus* (GMH-Leo VII-8004-1937, XXXVII-33-1964) actually falls within the size range of *P. hassiacum* (e.g., specimens GMH-XIV-2933-1956, XIV-3055, XIV-3027-1955).

(b) wedge-shaped cranium
*Comment*: Whether the skull shape of *E. parvulus* is more triangular (wedge-shaped) remains subjective without quantitative studies as the differences are otherwise not apparent. Moreover, skull shape allometry would be a plausible alternative for explaining shape differences.

(c) more slender postcranial skeleton
*Comment*: The proposed more slender postcranial skeleton of *E. parvulus* cannot be verified since the only skull-postcranial association known from Geiseltal (GMH-XXXVII-33-1964) is incomplete and strongly deformed. What is preserved (humerus, radius, some metacarpals, femur, pelvis and vertebrae) does not appear to be qualitatively more slender compared to specimens reffered to *P. voigti* (GMH XXXVII-135) and *P. isselanum* (GMH-Ce IV-7011-1933; no articulated postcranial material is preserved of *P. hassiacum*).

(d) sharper, less bulbous mesostyls on the upper molars and M3 as long as or shorter than M2

*Comment*: The less bulbous mesostyls on the upper molars in the specimen of *E. parvulus* (GMH-Leo VII-8004-1937, XXXVII-33-1964) are due to ontogeny as these apparently belong to young individuals based on their minimal tooth macrowear. Unsurprisingly, the mesostyle is blunter in most (larger) specimens referred to *P. hassiacum* with more worn molars, most likely representing older individuals. However, small specimens referred to *P. hassiacum*, (e.g., GMH-XIV-1379-1955, XIV-1311-1957, XIV-4007-1956) have mesostyls just as sharp as that of *E. parvulus*.

(e) Franzen and Haubold[6] further noted that subtriangular premolars without mesostyls are also diagnostic characters but these were subsequently omitted from the emended diagnosis of *E. parvulus*[6]
*Comment*: GMH-Leo VII-8004-1937 of *E. parvulus* may be more triangular than some *P. hassiacum* but clearly falls within the variation seem in *P. hassiacum* (e.g., GMH-XIV-1585-1954, XIV-799-1956, XIV-2235-1955). In addition, the premolars of *P. hassiacum* also lack a mesostyle.

## ***Invalid Taxa***

*Propalaeotherium voigti* Matthes 1977 (junior synonym of *P. isselanum*)

Type material: GMH XXXVII-135-1964, partial skeleton
Type locality: Geiseltal, XXXVII site, UMC
*P. voigti* was considered a small-sized, gracile species of *Propalaeotherium*[6,7]. The majority of specimens, including the type, are from Geiseltal but few have been reported from Messel and Eckfeld[8] as well. Our revision of the type material and the referred specimens from Geiseltal reveal that this is another taxon without diagnostic features and the characteristics listed by Franzen and Haubold[6] are also present in Geiseltal specimens referred to *P. isselanum* or *P. hassiacum*. We consider *P. voigti* as invalid and most likely synonymous with *P. isselanum*. Franzen and Haubold[6] considered the following characters diagnostic for *P. voigti*:

(a) skull length of adult individuals: 150-170 mm; skeletal mass 30% bigger than *E. parvulum* and 22-37% smaller than *P. hassiacum*+*P. isselanum*.
*Comment*: Franzen (Fig. 7–8 in ref. 8) himself illustrates that the size of the radius referred to *P. voigti* fall within the variation seen in *P. hassiacum* from Geiseltal. The skull size falls within the variation seen in *P. isselanum*.

(b) the molars show a clear increase in size (in the distal direction), while molars of *P. hassiacum*+*P. isselanum* become smaller
*Comment*: This character is evident in the holotype of *P. voigti* but less so in the referred specimen GMH-XXXVII 60. More importantly, it is also present in many specimens referred to *P. hassiacum* from Geiseltal (GMH-XIV 3238, XIV 1661, XIV 181) and can even be asymmetric within a single specimen (GMH-XIV 3645 and XIV 1585; left P2-P3 subequal in size, right side P3>P2). A distal size increase is present in specimens referred to *P. isselanum* as well (GMH-VI 574, XXXVI 280).

(c) lean body with short legs (similar to E. parvulum and different from *P. hassiacum* + *P. isselanum*)
*Comment*: Only two articulated *Propalaeotherium* skeletons are known from Geiseltal, one is the holotype of *P. voigti* (GMH-XXXVII-135-1964), a posteriorly incomplete skeleton with missing hind limbs whose body length is therefore unclear. The complete skeleton of *P. isselanum* GMH-Ce IV-7011-1933 is indeed more robust but the limb proportions appear to be near identical

to that of the holotype of *P. voigti*. The only complete skeleton referred to *P. voigti* comes from Messel as recently reported by Franzen[11]. Franzen does not discuss whether limb proportions are diagnostic for *P. voigti* but he does emphasize (and his Fig. 12.9.6 clearly demonstrates) that *P. voigti* is more gracile though only slightly smaller than *P. hassiacum*. In any case, demonstrating that *P. voigti* is proportionally consistently distinct from *P. isselanum* would require more than one complete referred skeleton of *P. voigti*.

(d) tooth mass is generally larger than *E. parvulum* and smaller than both *P. hassiacum* and *P. isselanum*; maxillary molars are mesially more stretched and shorter than *P. hassiacum* + *P. isselanum*
*Comment*: These differences are not quantified and therefore impossible to reproduce.

***(ii.2) Tapiromorpha***
***Valid Taxa***

TAPIROMORPHA Hooker, 1984
*LOPHIODON*TIDAE Gill, 1872
*Lophiodon remensis* Lemoine 1878

Four species of *Lophidon* have been reported from Geiseltal, including *Lophiodon tapirotherium* Desmarest, 1822, *L. cuvieri* Filhol, 1888, *L. remensis* Lemoine, 1878 and *L. buchsowillanum* Desmarest, 1822 (ref. 3,4). The most recent taxonomic revisions of Geiseltal *Lophidon* is Fischer[3] and it includes an emended diagnoses of the above species based on material from Geiseltal. The type species of the genus was designated as *L. tapirotherium* by Fischer[3] but he subsequently assigned this status to *L. tapiroides* Cuvier, 1912 and referred all material previously identified as *L. tapirotherium* from Geiseltal to *L. remensis*[12] . *L. remensis* is thus considered the most common species of *Lophiodon* in Geiseltal. It is beyond the scope of our work to verify this identification and we simply test whether the diagnoses of Fischer[4,12] are reproducible or, in other words, is there evidence for more than one species of *Lophiodon* at Geiseltal? The diagnoses of Fischer consists of qualitative, continuous characters and he apparently ignored intraspecific variation and preservational difference. We find that none of his differentiating characters can be objectively reproduced and we therefore recognize a single species at Geiseltal that we preliminarily refer to *L. remensis* but this identification is merely practical given that this is the conventional name used for the most abundant *Lophiodon* "species" at the locality[12] . Whether *L. remensis* is the correct name for the Geiseltal *Lophiodon* needs to be tested through comparison with the holotype, which we did not study.

***Dubious Taxa***

*Lophiodon buchsowillanum* Desmarest 1822

We are unable to distinguish *L. buchsowillanum* from *L. remensis* at Geiseltal because individuals of both taxa share the alleged differences. Fischer[4] listed the following characters to distinguish *L. buchsowillanum* from *L. remensis*:
(a) tooth row without diastema
*Comment*: A diastema is also present in *L. remensis* but in the few specimens available it is indeed shorter than the specimens referred to *L. buchsowillanum* by Fischer[4]. However, other species show quite some variation. This character is likely correlated with the size of the lower

canines and perhaps represents sexual dimorphism.

(b) shortened snout anterior to canines
*Comment*: This is likely correlated with the short diastema (see above). Moreover, all the specimens previously referred to *L. buchsowillanum* have incomplete snouts.

(c) narrow anterior end of nasals
*Comment*: the few specimens of the "species" where this region is preserved show identically narrow nasals.

(d) narrower apertura nasalis
*Comment*: We think this means a less concave margin of the antero-lateral portion of the nasal (i.e. nasals are more or less straight with parallel lateral sides). Few specimens of *Lophidon* from Geiseltal preserve this region but the character is nevertheless variable in *L. remensis*: in GMH-VII 338 1951 the antero-lateral margin of the aperture is concave whereas in GMH-LI 3856 the nasals are converging.

(e) Canines are small, not larger than the third incisor
*Comment*: The radius of the canine is indeed equal to the radius of the incisor in GMH-VI 43 of *L. buchsowillanum*. However, this condition is also seen in some *L. remensis* (e.g., GMH-VII 338-1951) and another *L. buchsowillanum* specimen (GMH-XXXVIII-69-1964) has very large canines relative to its incisors.

(f) Mesostyle absent, labial and lingual cingulum in upper molars absent
*Comment*: The mesostyle is present in GMH-XXXV-82d of *L. buchsowillanum* and absent in Y-16 of *L. remensis*, which demonstrates that the character is variable. The absence of labial and lingual cingulum in the upper molars is actually the usual condition for *Lophiodon* from Geiseltal except for a very few specimens.

(g) Upper premolars only with an inner cusp, which probably represents the fused protocone and hypocone. From this proto-hypocone, transverse v-shaped (not fully developed) ridges extend labially in P3 and P4.
*Comment*: This character is also present in *L. remensis* and only individuals with more worn teeth appear different.

(h) P2 has a ridge-shaped weak inner buldge; distinct metaconule in P3 and P4
*Comment*: as best seen in GMH-VI-43-1949 of *L. remensis*, the lingual face of the metacone has a ridge-like swelling and P3-P4 have distinct metaconules but both these characters are also present in *L. remensis* (e.g., GMH-10509-8).

(i) Lower molars typically rhinocerotoid by a very strong hypoconid front ridge, M3 hypoconulid weaker
*Comment*: the crista anterior to the hypoconid is actually also strongly developed in *L. remensis* and cannot be distinguished from the condition seen in *L. buchsowillanum*. The M3 hypoconulid is also similarly weakly developed in *L. remensis*.

(j) Lower premolars are without entoconid and the talonid is incomplete
*Comment*: In GMH-VI-45 of *L. buchsowillanum* the entoconid is indeed weakly developed because of tooth macrowear but other individuals have a clear entoconid on the premolars with a fully developed talonid.

*Lophiodon cuvieri* Filhol 1888

Fischer[12] synonymized *L. buchsowillanum* of Fischer[4] with *L. cuvieri* but kept the diagnosis from Fischer[4] . The diagnosis included the following characters: moderately large species; upper teeth are rounded and bulky; reduced cingulum; upper molar with nearly tapiroid metacone, parastyle appears rounded and accomodates the paracone. None of these characters are discretized nor quantified which precludes objective reproduction and we were unable to identify these differences compared to specimens of *L. remensis*. The only discrete character is the presence of enamel folds at the lingual base of the incisors but among *L. cuvieri* only GMH-XXXVIII-69-1964 preserves this region and such folds are likewise apparent in some specimens of *L. remensis*.

### (iii) References for Figure 5

Surface temperature data for the Eocene Central Europe is based on reconstructions from paleofloras[13,14,15,16,17], vertebrate oxygen isotopes[18] and peat GDGTs[19]. The global temperature curve is taken from Hansen et al.[20]. MAP estimates are derived from many of the same record[14,15,16,17] but also include simulated early-middle Eocene climates[21,22].

### (iv) Supplementary Figures S1-S4

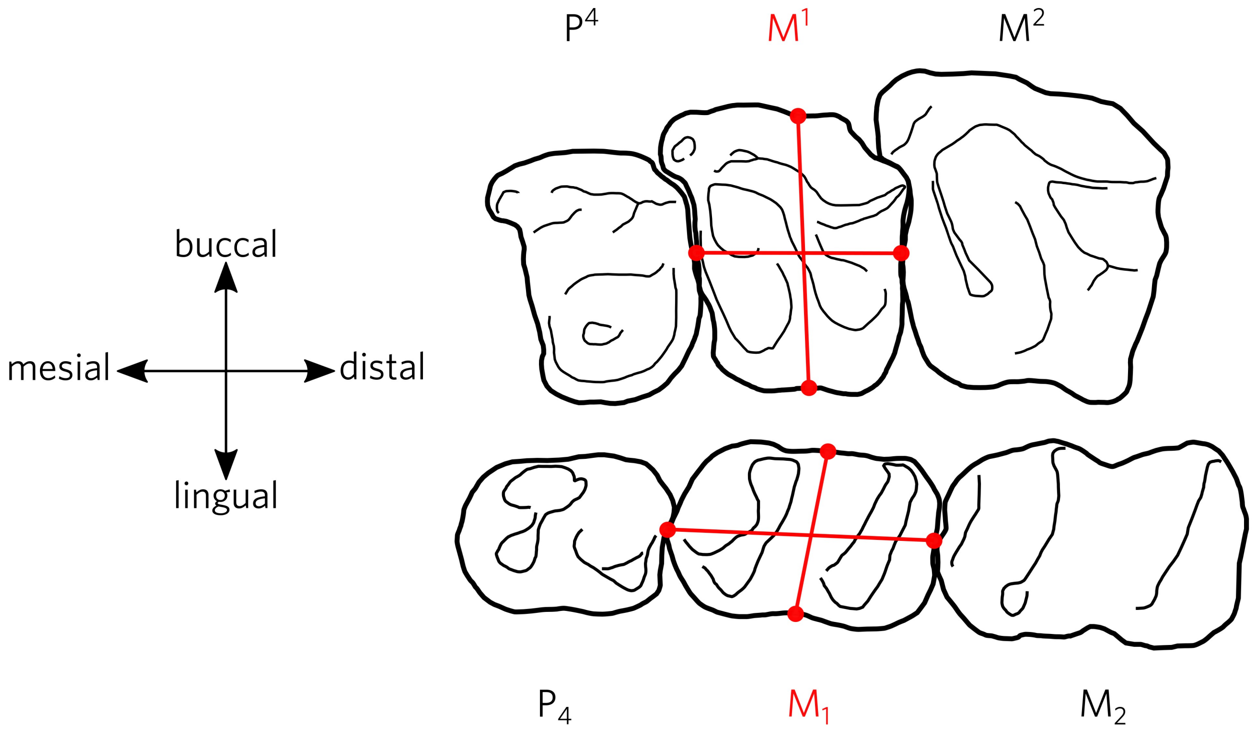


**Figure S1 | Schematic representation of upper (top) and lower (bottom) ungulate P4 through M2.** Red lines show an example of how a caliper measurement on the M1 would have been taken. Isolated teeth were excluded in our analysis because their original position within the tooth row could not be unambiguously identified.

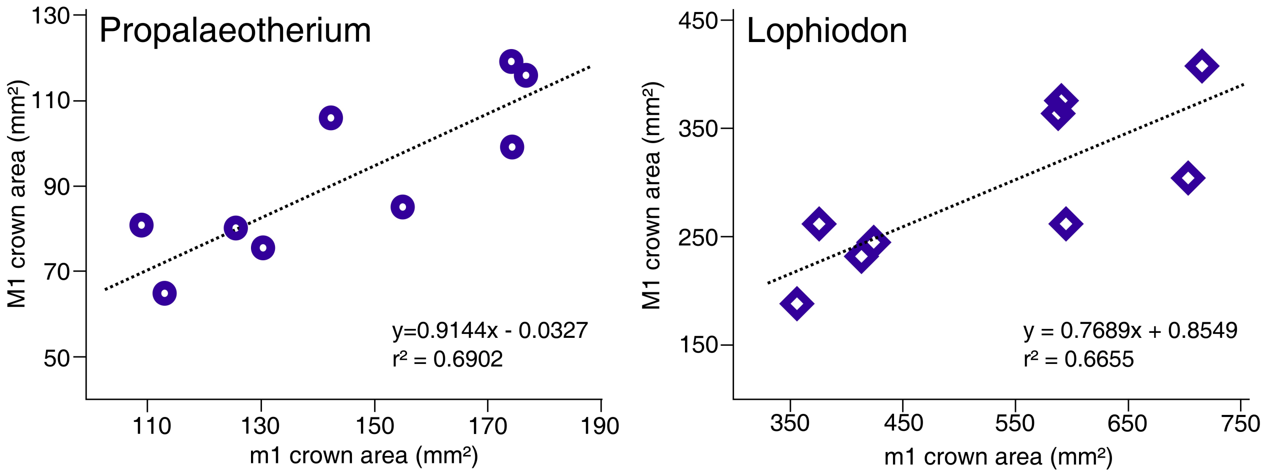


**Figure S2 | Tooth crown area regressions to predict m1-normalized body mass for both analyzed genera.** The regressions were constructed by measuring associated specimen where upper and lower teeth rows of a single individual are clearly preserved.

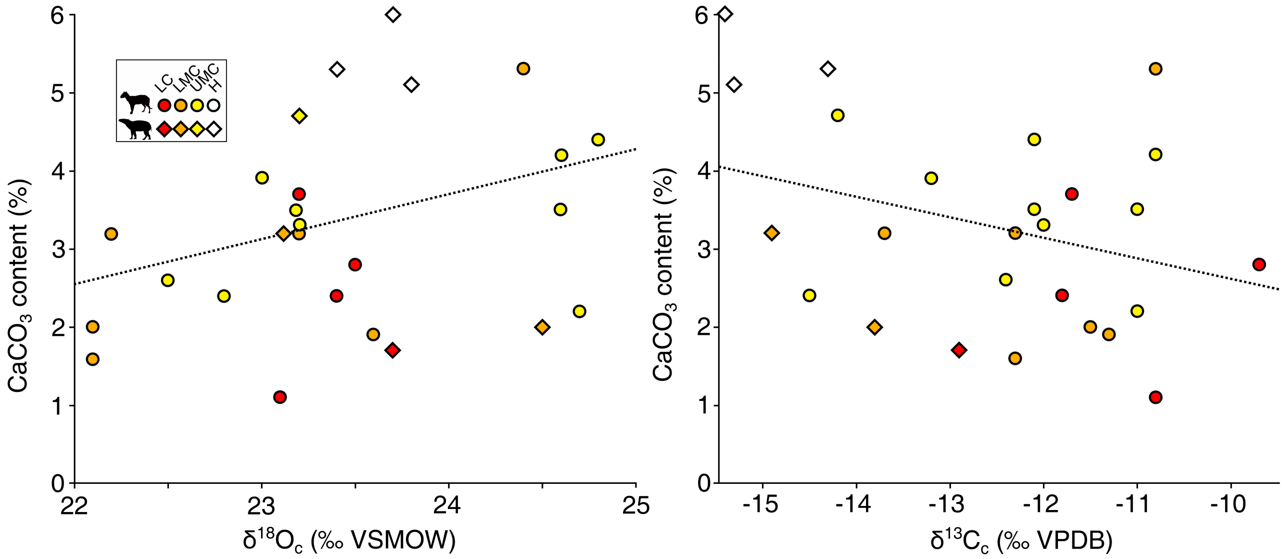


**Figure S3 | Variations in stable oxygen (left) and carbon (right) against carbonate content by weight (in %) and color-coded according to stratigraphic position.** Linear regression yields correlation coefficients ($r^2$) of 0.115 and 0.148, respectively. The extremely high scatter and lack of a clear correlation between isotopic measurements and calcification indicates that post-mortem of an isotopic signal through carbonate precipitation is not a significant mechanism in Geiseltal.

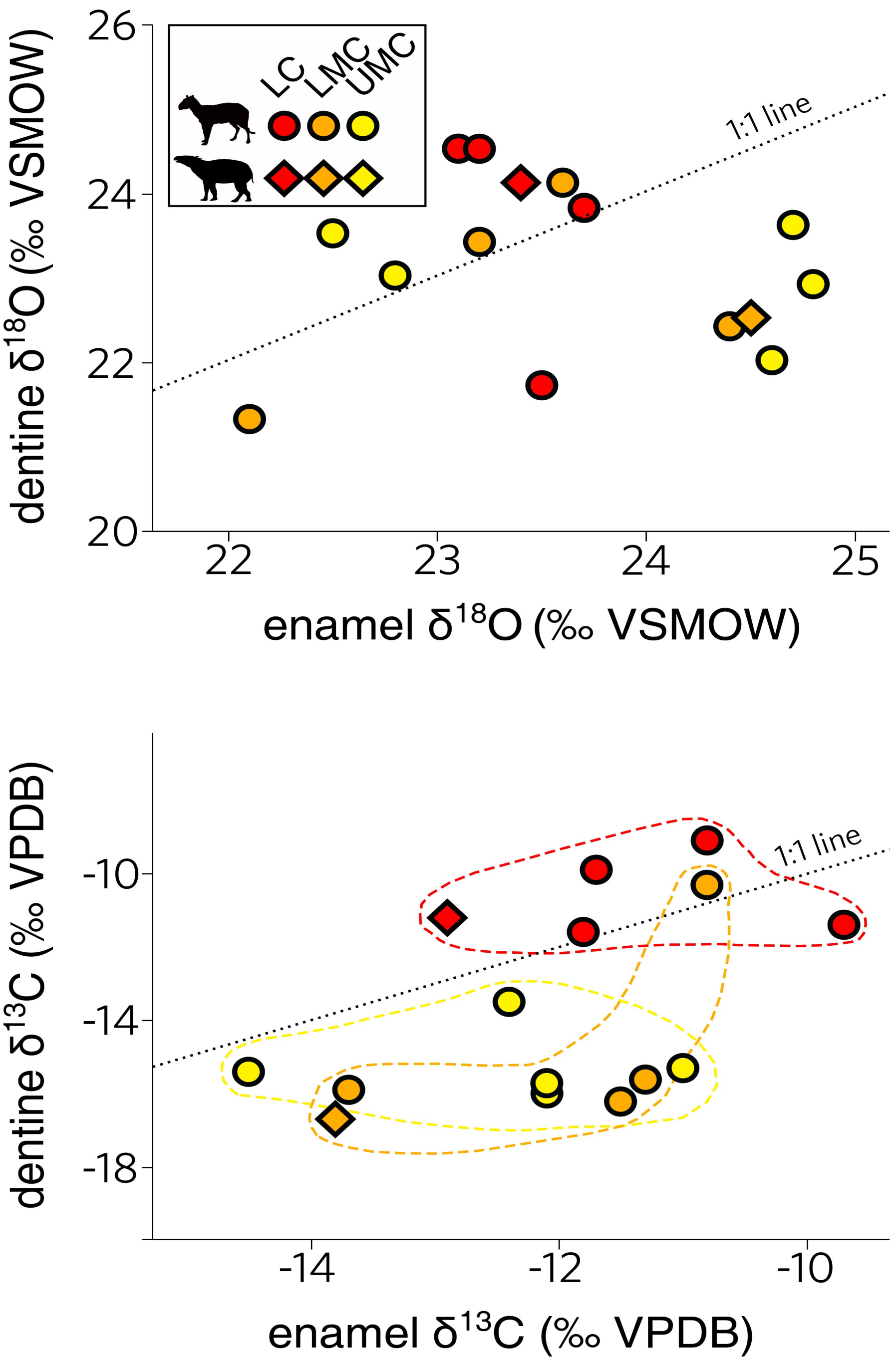


**Figure S4 | Reconstructed isotopic composition of enamel and detine for 15 samples where both values are known.** Other than in oxygen isotopes, $\delta^{13}C$ data points partially self-organize according to stratigraphic position. We interpret this as a progressive reduction in the influx of dissolved Triassic bicarbonate, which can help to explain the distribution of the Geiseltal fossil record as bicarbonate is considered to be a crucial mechanism for fossil preservation in peat bogs.

**Supplementary References**

1. Richard, M. Contribution à l'étude du Bassin d'Aquitaine: les gisements de mammifères tertiaires (in French). *Societé géologique de France* **24**, (1946).
2. Remy, J. A. Sur le crâne de *Propalaeotherium* isselanum (Mammalia, Perissodactyla, Palaeotheriidae) de Pépieux (Minervois, Sud de la France) (in French). *Geodiversitas* **23**, 105–127 (2001).
3. Fischer, K. H. Die tapiroiden Perissodactylen aus der eozänen Braunkohle des Geiseltales (in German). *Geologie* **45**, 1–101 (1964).
4. Fischer, K. H. Neue Funde von Rhinocero*lophiodon* (n. gen.), *Lophiodon*, und Hyrachyus (Ceratomorpha, Perissodactyla, Mammalia) aus dem Eozän des Geiseltals bei Halle (DDR). 1. Teil: Rhinocero*lophiodon*. *Zeitschrift für geologische Wissenschaft, Berlin* **5**, 909–919 (1977).
5. Wilde, V. & Frankenhäuser, H. The Middle Eocene plant taphocoenosis from Eckfeld (Eifel, Germany). *Review of Palaeobotany and Palynology* **101**, 7–28 (1998).
6. Franzen, J. L. & Haubold, H. Revision der Equoidea aus den eozänen Braunkohlen des Geiseltales bei Halle (DDR) (in German). *Palaeovertebrata* **16**, 1–34 (1986).
7. Haupt, O. Die Paläohippiden der eocänen Süsswasserablagerungen von Messel bei Darmstadt (in German). *Abhandlungen der hessischen geologischen Landesanstalt* **6**, 1–159 (1925).
8. Franzen J. L. *Eurohippus* parvulus parvulus (Mammalia, Equidae) aus der Grube Prinz von Hessen bei Darmstadt (Süd-Hessen, Deutschland) (in German). *Senckenbergiana lethaea* **86**, 265–269 (2006).
9. Hellmund, M. Odontological and osteological investigations on propalaeotheriids (Mammalia, Equidea) from the Eocene Geiseltal Fossillagerstätte (Central Germany) – a full range of extraordinary phenomena. *Neues Jahrbuch für Geologie und Paläontologie – Abhandlungen* **267**, 127–154 (2013).
10. Franzen, J. L. Report on the Discovery of Fossil Mares with Preserved Uteroplacenta from the Eocene of Germany. *Fossil Imprint* **73**, 67–75 (2017).
11. Franzen, J. L. Odd-toed ungulates - early horses and tapiromorphs. In: Smith, K. T., et al (Eds.) Messel – An ancient greenhouse ecosystem. *Senckenberg Gesellschaft für Naturforschung, Frankfurt am Main* (2018).
12. Fischer, K. H. Neue Funde von Rhinocero*lophiodon* (n. gen.), *Lophiodon*, und Hyrachyus (Ceratomorpha, Perissodactyla, Mammalia) aus dem Eozän des Geiseltals bei Halle (DDR). 2. Teil: *Lophiodon* (in German). *Zeitschrift für geologische Wissenschaft, Berlin* **5**, 1129–1152 (1977).
13. Pound, M. J. & Salzmann, U. Heterogeneity in global vegetation and terrestrial climate change during the late Eocene to early Oligocene transition. *Scientific Reports* **7**, (2017).
14. Moraweck, K., Uhl, D. & Kunzmann, L. Estimation of late Eocene (Bartonian–Priabonian) terrestrial palaeoclimate: Contributions from megafloral assemblages from central Germany. *Palaeogeography, Palaeoclimatology, Palaeoecology* **433**, 247–258 (2015).
15. Mosbrugger, V., Utescher, T. & Dilcher, D. L. Cenozoic continental climatic evolution of Central Europe. *Proceedings of the National Academy of Sciences of the United States of America* **102**, 14964–14969 (2005).
16. Grein, Michaela, Utescher, T., Wilde, V. & Roth-Nebelsick, A. et al. Reconstruction of the middle Eocene climate of Messel using palaeobotanical data. *Neues Jahrbuch für Geologie und Paläontologie – Abhandlungen* **260**, 305–318 (2011).
17. Krutzsch, W., Blumenstengel, H., Kiesel, Y. & Rüffle, L. Paläobotanische Klimagliederung des Alttertiärs (Mitteleozän bis Oberoligozän) in Mitteldeutschland und das Problem der Verknüpfung mariner und kontinentaler Gliederungen (in German). *N. Jb. Geol. Paläont. Abh.* **186**, 137–253 (1992).
18. Tütken, T. Isotope compositions (C, O, Sr, Nd) of vertebrate fossils from the Middle Eocene oil shale of Messel, Germany: Implications for their taphonomy and palaeoenvironment. *Palaeogeography, Palaeoclimatology, Palaeoecology* **416**, 92–109 (2014).
19. Inglis, G. *et al*. Mid-latitude continental temperatures through the early Eocene in western

Europe. *Earth and Planetary Science Letters* **460**, 86–96 (2017).
20. Hansen, J., Sato, M., Russell, G. & Kharecha, P. Climate sensitivity, sea level and atmospheric carbon dioxide. *Philosophical Transactions of the Royal Society A* **371**, (2017).
21. Carmichael *et al*. A model–model and data–model comparison for the early Eocene hydrological cycle. *Climate of the Past* **12**, 455–481 (2016).
22. Speelman, E. N., Sewall, J. O., Noone, D., Huber, M., von der Heydt, A., Damsté, J. S. & Reichart, G.-J. Modeling the influence of a reduced equator-to-pole sea surface temperature gradient on the distribution of water isotopes in the Early/Middle Eocene. *Earth and Planetary Science Letters* **298**, 57–65 (2010).
